# Accelerating genetic gains for quantitative resistance to verticillium wilt through predictive breeding in strawberry

**DOI:** 10.1002/tpg2.20405

**Published:** 2023-11-14

**Authors:** Mitchell J. Feldmann, Dominique D. A. Pincot, Mishi V. Vachev, Randi A. Famula, Glenn S. Cole, Steven J. Knapp

**Affiliations:** ^1^ Department of Plant Sciences University of California Davis Davis California USA

## Abstract

Verticillium wilt (VW), a devastating vascular wilt disease of strawberry (*Fragaria* × *ananassa*), has caused economic losses for nearly a century. This disease is caused by the soil‐borne pathogen *Verticillium dahliae*, which occurs nearly worldwide and causes disease in numerous agriculturally important plants. The development of VW‐resistant cultivars is critically important for the sustainability of strawberry production. We previously showed that a preponderance of the genetic resources (asexually propagated hybrid individuals) preserved in public germplasm collections were moderately to highly susceptible and that genetic gains for increased resistance to VW have been negligible over the last 60 years. To more fully understand the challenges associated with breeding for increased quantitative resistance to this pathogen, we developed and phenotyped a training population of hybrids (n=564) among elite parents with a wide range of resistance phenotypes. When these data were combined with training data from a population of elite and exotic hybrids (n=386), genomic prediction accuracies of 0.47–0.48 were achieved and were predicted to explain 70%–75% of the additive genetic variance for resistance. We concluded that breeding values for resistance to VW can be predicted with sufficient accuracy for effective genomic selection with routine updating of training populations.

## Core Ideas

- For the second half of the 20th century, strawberries were cultivated with high levels of chemical inputs.
- Genetic gains for resistance to verticillium wilt (VW) have been low, or negative, since the 1960s.
- VW is heritable, and breeding values can be accurately predicted to improve populations and individuals.
- The genetic architecture of resistance is similar among modern hybrids, heirloom varieties, and crop wild relatives

**Abbreviations**
BLUP: best linear unbiased prediction
EMM: estimated marginal mean
GEBV: genome‐estimated breeding value
GBLUP: genomic best linear unbiased prediction
GS: genomic selection
LMM: linear mixed model
MAS: marker‐assisted selection
VW: verticillium wilt

## 1 INTRODUCTION

Verticillium wilt (VW) is a devastating vascular wilt disease of strawberry (*Fragaria* × *ananassa* Duchesne) caused by *Verticillium dahliae* Klebahn, a widespread soil‐borne pathogen.*V. dahliae* penetrates the root of the plant, invades the vascular system, and causes chlorosis, necrosis, and often death in susceptible genotypes (Thomas, 1932; Wilhelm et al., 1956). This pathogen has a broad host range and a wide geographic distribution and thrives in temperate environments where strawberries are commonly grown (Fradin & Thomma, 2006; Pegg, 1984). *V. dahliae* produces microsclerotia (melanized hyphae) that can persist in the soil for many years, which diminishes the impact of crop rotation and complicates management practices for reducing losses to the disease (Short et al., 2015).

Verticillium wilt was first discovered in cultivated strawberry in the 1930s (Thomas, 1932) and became an important breeding target in the early half of the 20th century. Since the 1960s, the most effective method for controlling *V. dahliae* has been the application of powerful soil fumigants that suppress pathogen growth and decrease losses to diseases caused by *V. dahliae* and other soil‐borne pathogens. Consequently, the search for natural sources of resistance and breeding for increased resistance to verticillium wilt has lagged behind (Pincot et al., 2020). The phase‐out of the fumigant chemical methyl bromide (MeBr) according to the United Nations' Montreal Protocol (Velders et al., 2007) led to VW resistance reemerging as a trait critical to the sustained commercial production of strawberry (Holmes et al., 2020). This pathogen's widespread nature and persistence make it a major threat to strawberries and many other crops (Song & Thomma, 2018; Uribe et al., 2014).

Heirloom strawberry cultivars and wild relatives have been identified as potential sources of novel favorable alleles; however, these accessions also contain a high frequency of unfavorable alleles that would impact several horticulturally important traits, such as yield, shelf‐life, fruit quality, and resistance to other diseases, relative to modern‐day cultivars (Bringhurst et al., 1968; Jiménez et al., 2022; Pincot et al., 2020). There is great interest in identifying the genetic basis of *V. dahliae* resistance to facilitate the efficient incorporation of novel alleles into breeding materials without diminishing horticultural value.

Recently developed genetic and genomic technologies (Bassil et al., 2015; Edger et al., 2019; Hardigan et al., 2020, 2021) have enabled researchers to study the genetic architecture underlying disease resistance caused by several pathogens in strawberry (Mangandi et al., 2017; Nelson et al., 2021; Pincot et al., 2018, 2020). Antanaviciute et al. (2015) described several resistance loci for VW; however, the combination of these loci being of minor additive effect and necessitating several to be stacked to reach moderate resistance limits their use of marker‐assisted selection (MAS). We hypothesized that genomic prediction (GP) methodologies might improve breeding strategies to improve VW resistance in strawberry above and beyond what is possible with MAS (Pincot et al., 2020) and previous investigations have either found no major‐effect loci (Pincot et al., 2018), or a few minor‐effect loci controlling VW resistance in strawberry (Antanaviciute et al., 2015; Cockerton et al., 2019), limiting the effectiveness of commonly used plant breeding strategies, such as MAS. The recent introduction of high‐density arrays anchored on a high‐quality octoploid reference genome (Hardigan et al., 2020, 2021) has expanded the possibilities of genome‐informed breeding strategies (Whitaker et al., 2020). Indeed, using these tools, Pincot et al. (2020) were able to obtain accuracies between 0.38 and 0.54 and accounted for nearly all of the additive genetic variation of VW resistance in a diverse population of accessions representative of the global *Fragaria* germplasm. Pincot et al. (2020) demonstrated the promise of genomic selection approaches while also revealing significant space for improvement. Specifically, while the population incorporated many elite breeding cultivars, it also included diverse accessions, such as heirlooms and *Fragaria* ecotypes, which may not be well suited for predicting the performance of elite *Fragaria* × *ananassa* lines. Moreover, while disease progression data was collected, only final time point values were used for publication.

**Core Ideas**

- For the second half of the 20th century, strawberries were cultivated with high levels of chemical inputs.
- Genetic gains for resistance to verticillium wilt (VW) have been low, or negative, since the 1960s.
- VW is heritable, and breeding values can be accurately predicted to improve populations and individuals.
- The genetic architecture of resistance is similar among modern hybrids, heirloom varieties, and crop wild relatives

Here, we expand and improve upon previous work by utilizing previously collected data on the diverse population in (Pincot et al., 2020) 386 diverse *Fragaria* accessions including elite and wild ecotypes (henceforth the Global population) and newly collected data on a breeding population composed of 564 *F*. × *ananassa* accessions derived from a factorial mating design between elite breeding material, heirlooms, and ecotypes (henceforth, the CA population). With these two populations, we used a combination of final time point disease severity score assessment and the area under the disease progression stairs (AUDPS) (Simko & Piepho, 2012), which incorporates disease phenotyping throughout the season and thus information about severity/rapidity of disease development. Within each population, we observed prediction accuracies ranging from 0.42 to 0.48, similar to those found by Pincot et al. (2020). Across populations, prediction accuracy was lower (0.28–0.33). Combining the data from these two populations resulted in an average increase in the cross‐population prediction accuracy (0.43–0.48) in a powerful argument for the utility of multiple populations/year in a genomic prediction model. The combined population seems to provide an advantage as a training population as you are able to achieve equal prediction accuracy compared to within‐population analyses and superior prediction accuracy compared to across population analyses. Our work suggests that genomic prediction strategies using a combination of gene bank germplasm and strongly connected breeding material to train models, most likely obtained over several years of field evaluations, are superior to strategies incorporating only diverse germplasm or narrow breeding populations.

## 2 RESULTS AND DISCUSSION

### 2.1 VW resistance is heritable, but the environment plays a non‐negligible role

**FIGURE 1:**
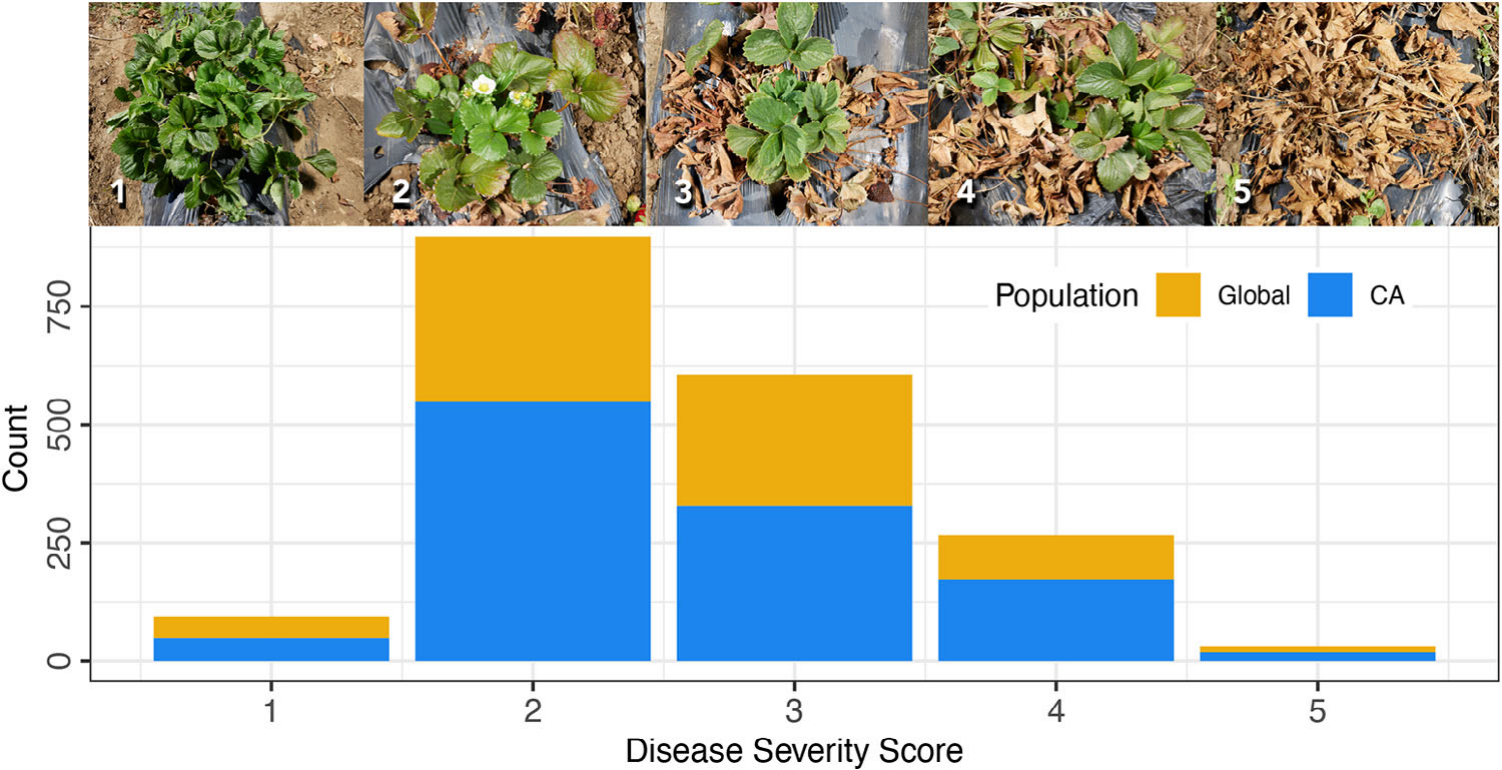
Verticillum wilt disease symptom rating scale (disease severity score) and phenotypic distributions for two training populations. The ordinal severity scores correspond to a progression of symptoms from symptomless (**1**) to complete plant collapse and death (**5**) and a chlorotic, wilted, and senescent leaf tissue increases from 2 to 5. The photographs are license‐free for UC Davis by Greaves Photography, July 2022, Davis, CA.

**FIGURE 2:**
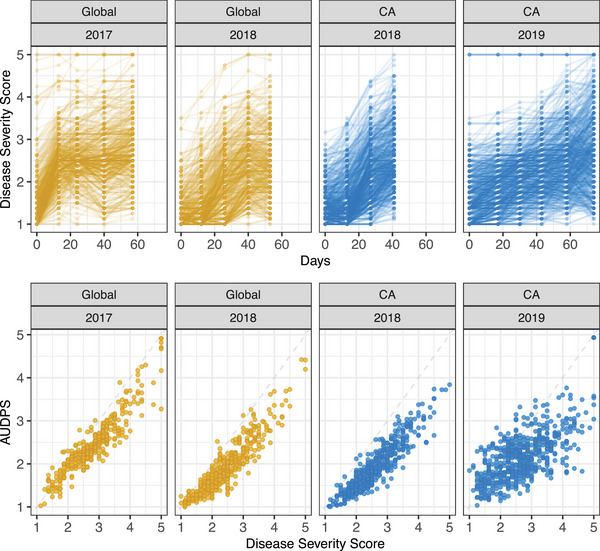
Verticillium wilt disease severity score and area under the disease progression stairs distributions are shown for individuals in Global (n=386; shown in gold) and CA (n=564; shown in blue) training populations screened in Davis, CA field experiments. The ordinal disease severity score rating scale was 1 = highly resistant, 2 = moderately resistant, 3 = moderately susceptible, 4 = susceptible, and 5 = highly susceptible. VW disease severity scores were recorded over four to six time points across the four experiments (39,124 phenotypic observations among 921 individuals). The upper panel shows the progression of symptoms (change in disease severity score) from the onset (day zero) to the final time point in each experiment.

**FIGURE 3:**
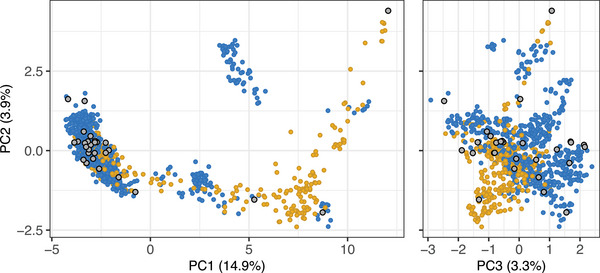
Principal component analysis (PCA) reveals significant population structure in Global and CA populations. PCA of the genotypes of the accessions in the Global (n=358; shown in gold), CA (n=534; shown in blue), and both (n=28; shown in gray) experimental populations. Numbers in parentheses on axis labels represent the percent variation explained by each principal component. The individuals in both experiments comprise 28 of 30 hybrid parents of the CA populations. Genomic PCA is only shown for those samples with genotypic data.

We observed substantial phenotypic variability for VW resistance in both the Global and CA populations (Figures 1, 2, 3). Phenotypic and genotypic data are provided in Files S1 and S2. The within‐ and between‐year estimated marginal means (EMMs) for the score at the final time point were approximately normally distributed for each experiment, and the EMMs spanned the entirety of the ordinal scale (1 = asymptomatic, 5 = dead) (Figure 1). Likewise, year‐to‐year variation has also been described in other crops, with significant genotype‐by‐environmental/year effect detected in lettuce and sunflower (Montecchia et al., 2021; Sandoya et al., 2021). The (EMMs for AUDPS (Simko & Piepho, 2012) also appeared normally distributed, though with highly susceptible outliers causing the distribution to be skewed right. In general, the scores in 2017 (Global population) and 2019 (CA population) were more severe than those in 2018 when both populations were evaluated.

The impact of G×E is evident from our analyses (Table 1). Score and AUDPS were highly correlated for each population in individual years ($r_{\text{Global}}=0.87-0.91$; $r_{\text{CA}}=0.69-0.89$), the phenotypic correlations for VW resistance across years of testing tended low‐to‐moderate and were reduced in the CA population ($r_{\text{Score}}=0.15$; $r_{\text{AUDPS}}=0.14$) compared to the Global population ($r_{\text{Score}}=0.54$; $r_{\text{AUDPS}}=0.50$; Figure 2). This could be in part caused by the different experiment lengths between two years of CA trials and it is possible that the 2018 experiment (40 days) is too different from the 2019 experiment (70 days). The impact of G×E was also reflected in broad‐sense and narrow‐sense heritabilities estimates. When we estimated single‐year broad‐sense heritabilities ($H^{2}$) and narrow‐sense heritabilities ($h^{2}$) of the Score and AUDPS traits in both populations, we found moderate values for both ($H^{2}=0.35-0.68$; $h^{2}=0.33-0.49$; Table 1). In general, the traits showed higher broad‐sense and narrow‐sense heritabilities in the Global population than in the CA population. As with the phenotypic correlation, the between‐year estimates of $H^{2}$, but not $h^{2}$ were greatly reduced in the CA population ($H^{2}=0.54-0.6$; $h^{2}=0.31-0.43$) and not in the Global population ($H^{2}=0.60-0.73$; $h^{2}=0.36-0.49$) (Table 1). Clones performed and were scored similarly within and between years in the Global population ($\sigma_{G\times E}=7\%-13\%$). Conversely, more significant variation was observed in the performance of clones across years in the CA population ($\sigma_{G\times E}=21\%-32\%$). G×E also impacted the AUDPS trait more than the Score trait. AUDPS, by its essence, is a trait linked to the time over which the experiments were performed, encapsulating environmental and seasonal variables such as changes in temperature, moisture, and so on, which may have contributed to the more significant impact of G×E.

**TABLE 1:** Restricted maximum likelihood estimates of broad‐sense heritability ($H^{2}$), narrow‐sense heritability ($h^{2}$), the proportion of the phenotypic variance explained by genotype‐by‐environmental variance ($\sigma_{G\times E}^{2}/\sigma_{P}^{2}$), the square root of the additive genetic variance ($\sqrt{\sigma_{A}^{2}}$), and the coefficient of additive genetic variation (${\text{CV}}_{A}$) for VW disease severity score and area under the disease progression stairs (area under the disease progression stairs) among individuals in global and CA populations. Genomic‐estimated heritability and additive genetic variance ($h^{2}$, $\sqrt{\sigma_{A}^{2}}$, and ${\text{CV}}_{A}$) were estimated using only those individuals with both phenotypic and genotypic data in the global (n=386), CA (n=564), and global + CA (n=921) populations.

Trait	**Population** **(** ***n*** **)**	Year	$H^{2}$	$\sigma_{G\times E}^{2}/\sigma_{P}^{2}$	$h^{2}$	$\sqrt{\sigma_{A}^{2}}$	${\text{CV}}_{A}$
Score	Combined (921)	Combined	0.46	0.21	0.42	0.40	0.15
		2018	0.60	—	0.40	0.45	0.18
	CA (564)	Combined	0.35	0.27	0.34	0.37	0.14
		2018	0.58	—	0.33	0.40	0.16
		2019	0.54	—	0.43	0.60	0.21
	Global (386)	Combined	0.68	0.07	0.47	0.43	0.15
		2017	0.65	—	0.36	0.42	0.14
		2018	0.60	—	0.43	0.48	0.19
**AUDPS**	Combined (921)	Combined	0.50	0.23	0.48	0.33	0.14
		2018	0.61	—	0.40	0.35	0.18
	CA (564)	Combined	0.34	0.32	0.33	0.25	0.14
		2018	0.59	—	0.31	0.30	0.16
		2019	0.61	—	0.36	0.36	0.16
	Global (386)	Combined	0.66	0.13	0.49	0.36	0.14
		2017	0.73	—	0.47	0.41	0.16
		2018	0.63	—	0.41	0.38	0.20

### 2.2 Potential donors of favorable alleles for enhancing quantitative resistance to verticillium wilt

Our study indicates that strong VW (disease score ≤1.5) resistance is rare in the broader germplasm of strawberry (≤8.5% of assessed accessions), is significantly impacted by environmental conditions, and shows quantitative resistance, unlike other soil‐borne diseases, for example, the FW loci for Fusarium wilt, the FaRPc2 locus for Phytophthora crown rot (PhCR), the MP loci for charcoal rot (Jiménez et al., 2022; Mangandi et al., 2017; Nelson et al., 2021; Pincot et al., 2022). In 2018 and 2019, respectively, 17.70% and 20.28% of accessions in the CA population were deemed resistant. In 2017, 12.95% of accessions in the Global population were resistant, whereas 27.46% were resistant in 2018. A more in‐depth examination of the Global population can be found in Pincot et al. (2020). When considering the overall performance across both populations and all years, 22.83% of accessions were resistant across both populations and all years. Thirty accessions were sampled in both elite and Global populations, and only two accessions, ‘08C128P002‘ and ‘Puget Reliance‘ (PI664321), were resistant across all years. As with PhCR and Fusarium wilt (FW) (Jiménez et al., 2022; Pincot et al., 2022), individuals carrying novel resistance alleles may exist in underutilized gene bank collections at low allele frequencies, decreasing the likelihood of detection and introgression. Along with the complex inheritance model, genetically resistant individuals likely carry many favorable alleles, each with small, statistically undetectable effects outside the most targeted (biparental mapping) or largest (n>>1000) populations (Yengo et al., 2022). The main challenge is that no large effect loci conferring resistance to VW have been identified in strawberry, leading us to hypothesize that (1) effect sizes are very small or (2) beneficial allele frequencies are exceedingly rare.

### 2.3 Multivariate GWAS further substantiates the complex genetic architecture of resistance to verticillium wilt in strawberry

**FIGURE 4:**
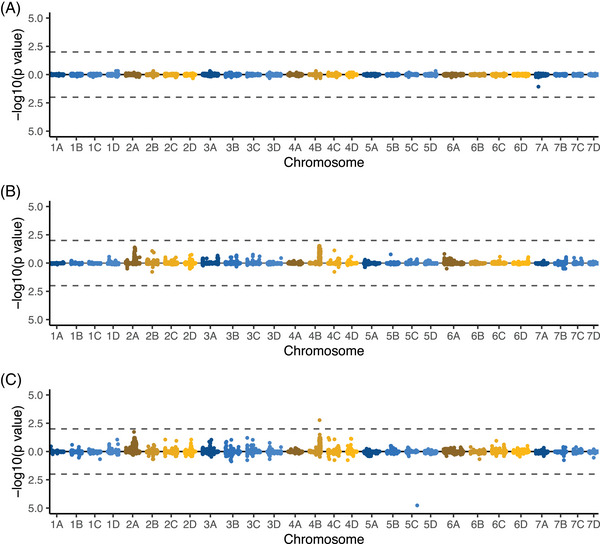
Multi‐trait genome‐wide association studies reveal minor effect loci on chromosomes 2A and 4B Manhattan plots of marker‐trait associations for VW disease severity score in the Global (n=373; **A**), CA (n=559; **B**) and combined (n=921; **C**) populations. For each population, the association results for the ordinal score at the final time point and the area under the disease progression stair (AUDPS) (Simko & Piepho, 2012) are shown above and below the y=0 line, respectively. Results shown are corrected at a false discovery rate (FDR) of 0.05, and the horizontal dashed lines signify an FDR threshold of p=0.05 ($-\log_{10}\left(0.05\right)=2.0$). Chromosome and base pair positions were obtained from the “UCD Royal Royce” FaRR1 V1.0 reference genome (Hardigan et al., 2021).

To make the most of both previously reported data (Pincot et al., 2020), as well as data for additional years and populations, we conducted a multi‐trait GWAS (MV‐GWAS) for both score and AUDPS, in which the EMMs for score and AUDPS from the multiple years of testing are treated as correlated traits (Zhou & Stephens, 2014). GWAS results are provided in File S3. We did not identify any significant peaks for AUDPS or score in the Global or CA panels at a significance threshold of $p_{\text{FDR}}=0.05$ (Supporting Information). These results aligned with the findings of (Pincot et al., 2020) and the hypothesis that VW resistance is a quantitative trait with complex inheritance. It is also possible that moderate‐to‐large effect favorable alleles do exist, but simply are not at high enough frequencies to be discovered by GWAS in our populations. MV‐GWAS of the combined population did identify two markers significant at an FDR threshold of $p_{\text{FDR}}=0.05$: for Score, AX‐184625933 on chromosome 4B and, for AUDPS, AX‐184699332 on chromosome 5C (Figure 4) (Supporting Information). In the combined analysis, the regression coefficients (marker effects) for AX‐184625933 were −0.20 and 0.41 (joint p value $=6.50e^{-8}$) for disease severity score, but no significant results for AUDPS (joint p value $=2.70e^{-1}$). Similarly, in the combined analysis, the regression coefficients for AX‐184699332 were −1.01 and 6.34 (joint p value $=6.62e^{-10}$) for AUDPS, but no significant results for disease severity score (joint p value $=1.06e^{-1}$). We were not able to discover/confirm these loci in any single population or single‐year analysis. Because marker effects are small and appear to switch signs, we do not believe these results are actionable in an MAS framework.

### 2.4 Genomic prediction predicts VW resistance with moderate accuracy

The effective use of genomic selection relies on retraining the statistical models by adding and removing data across breeding cycles. However, the addition of unrelated individuals has generally been shown to impact accuracy (Lorenz & Smith, 2015) with a few exceptions (Auinger et al., 2016). We used the breadth of the data in this experiment to estimate how genomic prediction would perform as new data is acquired and incorporated into the prediction model. The prediction accuracy for the combined population only moderately improved over the other training populations, with almost double the population size (Table 2). Prediction accuracies did not differ significantly between the three prediction models investigated (± 0.01) (Table 2).

**TABLE 2:** Genomic prediction accuracy: Accuracy of three whole‐genome regression methods for predicting verticillium wilt resistance in the diversity panel (n=373), California (CA) (n=559), and combined (n=921) populations.

Trait	Scheme	Train subset (*n*)	Test subset (*n*)	GBLUP	BRR	BL
Score	S1	80% Combined (737)	20% Combined (184)	0.47 ± 0.06	0.47 ± 0.06	0.47 ± 0.06
	S2	80% Combined (737)	20% CA (184)	0.43 ± 0.05	0.43 ± 0.05	0.43 ± 0.05
	S3	80% Combined (737)	20% Global (184)	0.48 ± 0.04	0.48 ± 0.04	0.48 ± 0.04
	S4	80% CA (452)	20% CA (112)	0.43 ± 0.07	0.43 ± 0.07	0.43 ± 0.07
	S5	80% CA (452)	100% Global (386)	0.37 ± 0.03	0.37 ± 0.03	0.37 ± 0.03
	S6	80% Global (309)	100% CA (564)	0.28 ± 0.02	0.28 ± 0.01	0.28 ± 0.01
	S7	80% Global (309)	20% Global (77)	0.48 ± 0.09	0.48 ± 0.09	0.48 ± 0.09
AUDPS	S1	80% Combined (737)	20% Combined (184)	0.45 ± 0.06	0.45 ± 0.06	0.45 ± 0.06
	S2	80% Combined (737)	20% CA (184)	0.47 ± 0.05	0.47 ± 0.05	0.47 ± 0.05
	S3	80% Combined (737)	20% Global (184)	0.44 ± 0.05	0.44 ± 0.05	0.44 ± 0.05
	S4	80% CA (452)	20% CA (112)	0.42 ± 0.07	0.42 ± 0.07	0.42 ± 0.07
	S5	80% CA (452)	100% Global (386)	0.33 ± 0.03	0.33 ± 0.02	0.33 ± 0.04
	S6	80% Global (309)	100% CA (564)	0.33 ± 0.01	0.33 ± 0.01	0.33 ± 0.01
	S7	80% Global (309)	20% Global (77)	0.43 ± 0.11	0.43 ± 0.11	0.44 ± 0.11

*Note*: The accuracy of Bayes ridge regression (BRR), Bayesian lasso (BL), and genomic best linear unbiased predictor (GBLUP) were estimated as the average correlation of predicted and observed phenotypes in 1000 Monte Carlo cross‐validations with accessions randomly assigned to training and test sets. VW resistance was scored on an ordinal scale (1 = healthy to 5 = dead). The estimated marginal means (EMMs) for disease severity scores at the final time point (score) and area under the disease progression stairs (AUDPS) estimates were used for genomic prediction.

We found that large populations with an abundance of additive genetic variance and a high degree of genomic connectedness make for superior training populations for genomic prediction (Kadam et al., 2021; Olatoye et al., 2020; Yu et al., 2017). For experiments using the CA training population to predict the Global panel, we may be dealing with an “out‐of‐bounds” prediction problem, as the genetic variation is much lower in the elite portion of the CA population than in the Global panel. On the other hand, diversity may not be a pure benefit. The experiments using the Global panel to predict the CA population were some of the least accurate, suggesting that unconnected diversity does not improve genomic selection (Daetwyler et al., 2012). The most promising experiments utilized both populations in the training data set (Table 2). We achieved prediction accuracies of 0.47 and 0.49 for Score and AUDPS phenotypes, respectively. Using the narrow‐sense heritability estimates (${\hat{h}}^{2}$) from Table 1 as a proxy, we estimate that our models explain 70%–75% of the additive genetic variance.

Within‐population prediction accuracy for Score and AUDPS were moderate for both populations: 0.42 and 0.42 for the CA population and 0.49 and 0.44 for the Global population for 2‐year EMMs. However, it should be noted that the two populations assessed in this study and that of Pincot et al. (2020) represent accessions separated by time, geography, and breeding efforts—one sampling within an elite Californian breeding population and the other sampling widely across the available *Fragaria* germplasm diversity worldwide. Even if it has been shown that allelic diversity has been maintained in octoploid strawberry, from the wild progenitor ecotypes to modern populations undergoing strong selection (Hardigan et al., 2021; Pincot et al., 2021), we still saw the accuracy of between‐population predictions decrease when compared to within‐population predictions (Table 2). Considering that our least effective prediction scenarios were between population, that is, schemes S5 and S6 (Table 2), the best strategy for implementing GP for this trait may incorporate smaller, targeted training populations with some optimized diversity, as opposed to large, global training population (Brandariz & Bernardo, 2019). Training population optimization for the specific target population is vital to achieving actionable and interpretable results (Kadam et al., 2021; Olatoye et al., 2020; Yu et al., 2017). It may prove challenging to develop an appropriately connected training population that can maintain prediction accuracy across years and pedigrees.

### 2.5 Simulations suggest that genomic selection may increase the quantitative resistance to VW with decreased variance over phenotypic selection

**FIGURE 5:**
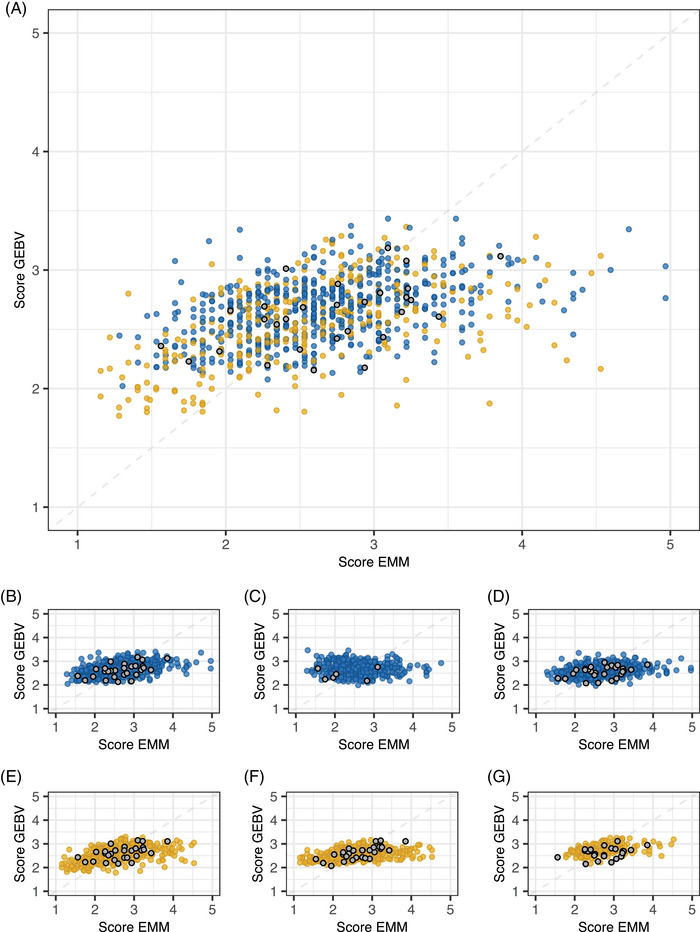
Disease severity score genomic‐estimated breeding values (GEBVs) plotted against the disease severity score estimated marginal means (EMMs). The EMMs and GEBVs for disease severity score are plotted for the 7 GS schemes evaluated. (**A**) Results from S1. (**B**) Results from S2. (**C**) Results from S4. (**D**) Results from S6. (**E**) Results from S2. (**F**) Results from S5. (**G**) Results from S7. In each scheme, 100 iterations were evaluated. Blue points are samples from the elite panel, gold points are from the Global panel, and grey points are observed in both panels. The light gray points in the figure backgrounds are the individual best linear unbiased prediction estimates from each CV iteration.

**FIGURE 6:**
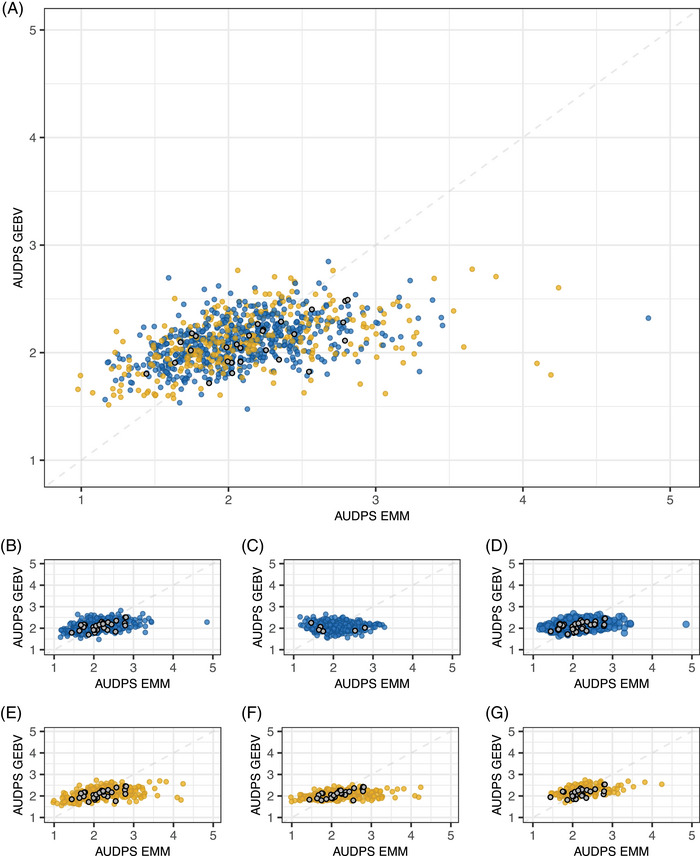
Area under the disease progression stairs (AUDPS) genome estimated breeding values (GEBVs) plotted against the AUDPS estimated marginal means (EMMs). The EMMs and GEBVs for the area under the disease progression stairs (AUDPS) are plotted for the 7 GS schemes evaluated. (**A**) Results from S1. (**B**) Results from S2. (**C**) Results from S4. (**D**) Results from S6. (**E**) Results from S2. (**F**) Results from S5. (**G**) Results from S7. Blue points are samples from the elite panel, gold points are from the Global panel, and grey points are observed in both panels. The light gray points in the figure backgrounds are the individual best linear unbiased prediction estimates from each CV iteration.

**FIGURE 7:**
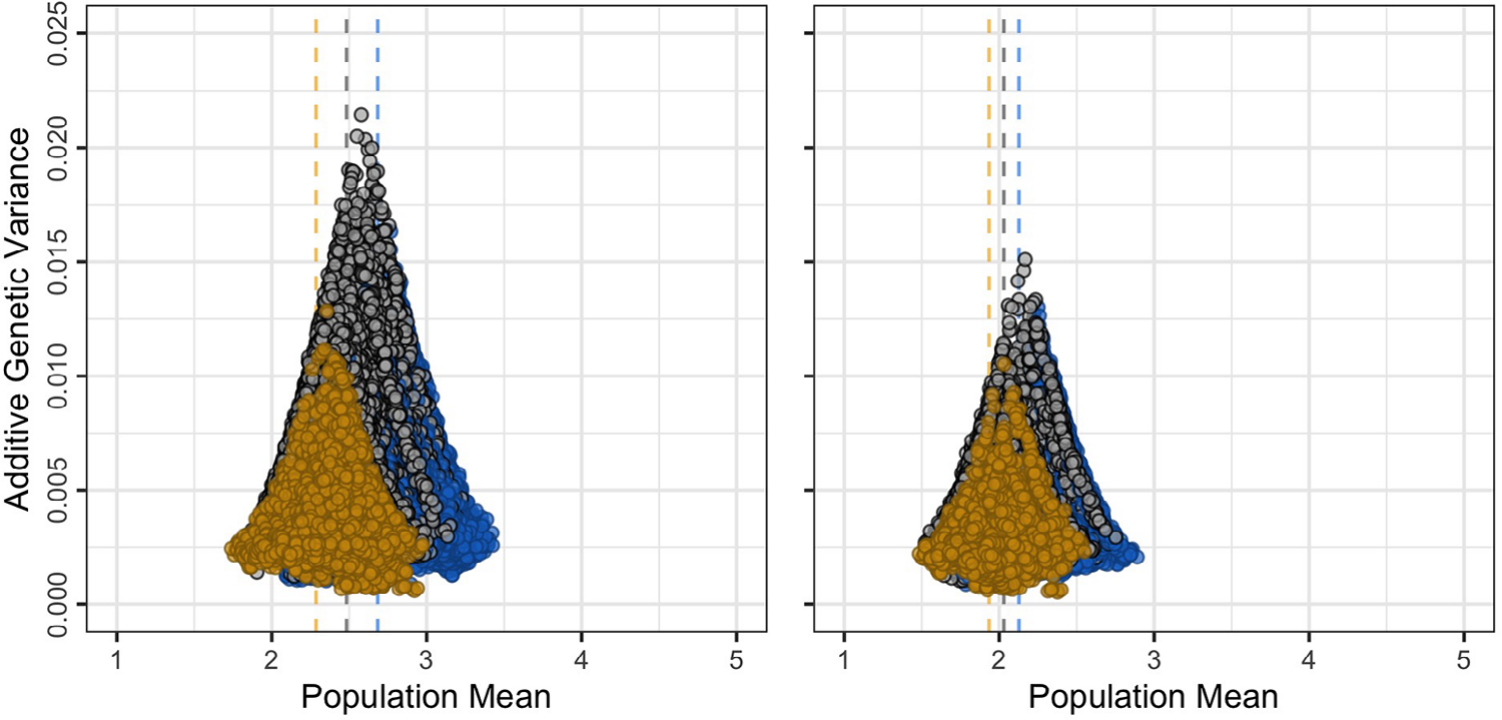
Heirloom varieties, wild accessions, and other exotic sources increase the average genetic resistance to verticillium wilt. All pairwise crosses (n=423,660) derived from the Global population and the CA populations were simulated. Predicted crosses were classified by the origin of parent ‘1‘ and parent ‘2‘, with designations being Exotic or UC Davis (UCD). There were 316,410 UCD x UCD crosses (blue), 99,500 UCD x Exotic crosses (gray), and 7750 Exotic x Exotic crosses (gold). The top row shows the cross‐usefulness criterion ($U={\hat{\mu }}_{g}-{\hat{\sigma }}_{g}$) for the disease severity score and area under the disease progression stairs (left to right). For visualization purposes, we show 75,000 of 316,410 UCD x UCD crosses, 25,000 of 99,500 UCD x Exotic crosses, and all 7750 Exotic x Exotic crosses to highlight the scores of the Exotic x Exotic crosses. The bottom row shows the predicted genetic mean by the predicted genetic variance for the disease severity score and AUDPS (left to right).

The accessions assessed by Pincot et al. (2020) and in this study covers a broad segment of octoploid *Fragaria* diversity; this includes heirlooms, ecotypes, and breeding lines for which VW resistance data has not been previously reported. These unobserved entries could potentially be available donors for VW resistance in public germplasm collections. The utilization of these accessions, many of which may have non‐viable commercial traits, would require several generations of hybridization or back‐crossing. This process could be accelerated through careful parent selection. We estimated the efficiency of genomic selection in selecting Verticillium resistance by simulating the mean and variance of hybrid families produced from all possible parental combinations in the elite and Global populations (n=423,660 total combinations). Each combination was categorized as resistant × resistant (R×R)—for both phenotype and genomic‐estimated breeding value (GEBV), a score <2 indicates resistance—resistant × susceptible (R×S), and susceptible × susceptible (S×S) depending on the observed phenotypes or the GEBVs of the parents involved. Using phenotypic observations resulted in 21,115 combinations categorized as R×R, whereas GEBVs categorized only 1596 combinations as such (Figures 5 and 6); the discrepancy arose mainly from material from the CA population. Simulations indicated that parental crosses ranked as R×R using GEBVs would outperform those ranked as R×R using phenotypic observations by an average of 0.47 ($p_{t}\leq 2.2\times 10^{-16}$). This strategy requires constant prediction validation to build internal and external confidence. The decreased mean and variance in the simulated families suggest that using GEBVs can help us to select individuals with better genetics to contribute to future generations (Figure 7). Therefore, we believe that genomic prediction can be used to predict winning entries as part of a product development pipeline and predict winning parents as part of the selection strategy in an efficient two‐part breeding program (Gorjanc et al., 2018).

## 3 CONCLUSIONS

Genetic gains for VW resistance have been negative over the past century, likely due to the historic widespread use of fumigants and prioritization of horticultural traits such as yield, fruit size, and firmness (Feldmann et al., 2023; Pincot et al., 2020). While promising resistance donors have been identified, they often carry unfavorable alleles for other target traits, leading to horticultural inferiority compared to modern cultivars. This challenges any breeder utilizing these donors to introduce multiple disease resistance alleles into elite genetic material. Genomic prediction could be an efficient breeding strategy for rapidly accumulating many beneficial alleles with minor effects. The best lines showing good VW resistance and horticultural performance could be targeted for advancement. Our study shows the viability and accuracy of genomic prediction within and between populations and the subsequent increase in prediction accuracy by combining data. Utilizing genomic prediction for selecting resistant entries and parents can make product advancement more time and cost effective. Therefore, we believe the genomic prediction approach could rapidly increase genetic gain for resistance and reverse the trend toward susceptibility established over the past century. Novel genetic and genomic technologies have supported genomic prediction as a burgeoning strategy that allows effective breeding for resistance through prediction models continuously updated with new genotypic and phenotypic data. As the prevalence of destructive plant pathogens continues to grow and impact strawberry productivity, adopting new technologies into the breeding toolbox would allow breeders to address the changing needs of the industry better and with more rapid responses. (Ramstein et al., 2019; Wallace et al., 2018).

## 4 MATERIALS AND METHODS

### 4.1 Plant material and clonal propagation

The populations we studied comprised 564 *F*. × *ananassa* accessions (individuals) developed by the University of California strawberry breeding program in a factorial mating design in 2016, referred to as CA hereafter, and 386 diverse *Fragaria* accessions evaluated as part of Pincot et al. (2020) (Supporting Information); referred to as Global hereafter. These populations are henceforth referred to as the elite and Global populations, respectively; for the latter, we utilized the genotypic and phenotypic data reported in Pincot et al. (2020) to perform cross‐population predictions with the CA population. The Global population was assessed in 2017 and 2018, and the CA population in 2018 and 2019. A combined population (n=921) comprising all entries from both populations was created for analysis.

We obtained source plants (clones) of the accessions of the CA population from the UCD Strawberry Germplasm Collection. Clonally propagated plants (daughter plants) of each accession were produced in high‐elevation (1294 m) nurseries in Dorris, CA, from mother plants produced in low‐elevation (41 m) nurseries in Winters, CA. The propagation of plant material was identical in both years (2018 and 2019): mother plants were harvested from the low‐elevation nursery in January, stored at $-3.5^{circ}$ C, and planted in the high‐elevation nursery in April. Clones were harvested in October and stored at 3.5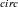 C for 2 to 3 weeks before pathogen inoculation and planting.

### 4.2 Pathogen source material and inoculum preparation

Vd9602, the virulent *V. dahliae* isolate used in our study, was originally isolated from strawberry plants growing in a commercial nursery in Macdoel, California (Gordon et al., 2006) and belongs to the 4B vegetative compatibility group (VCG) and II‐1 subclade (Fan et al., 2018; Milgroom et al., 2014; Pincot et al., 2020; Zeise & Von Tiedemann, 2002). VCG 4B isolates are often recovered from symptomatic strawberry plants in coastal California (Atallah et al., 2011; Bhat & Subbarao, 1999; Zeise & Von Tiedemann, 2002). The species‐specific sequence for the Vd9602 isolate can be found in GenBank (MN813047). To produce the inoculum for this experiment, the pathogen was first grown on potato dextrose agar under continuous fluorescent lighting at room temperature, as described in Shaw et al. (1996). Spores were freed from the surface by adding sterile, deionized (DI) water to the growth plates and scraping the medium with a sterile glass slide. The resulting suspension was filtered through two layers of sterilized cheesecloth to remove hyphae. Spore densities were estimated using a hemocytometer and diluted with sterile DI water to a final density of 1 × 10^6^ spores mL^‐1^. Spore suspensions were prepared 1 day before planting and stored overnight at 4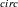 C. The suspension was shaken to re‐suspend spores before inoculating bare‐root plants.

### 4.3 Field experiments

Accessions were grown and phenotyped for resistance to VW in field experiments at the UCD Plant Pathology Research Farm in Davis, CA, where the soil type is a Yolo loam (https://websoilsurvey.sc.egov.usda.gov/). Strawberries had not been previously grown on the field sites selected for our studies. Site preparation, inoculation of plants with the pathogen, planting, fertilization, irrigation, and other management practices were identical in both years. Fields were pre‐plant flat fumigated with a chloropicrin‐based fumigant (Pic‐Clor 60, Cardinal Professional Products, Woodland, CA) at 500 lb acre^‐1^ and sealed with an impermeable film tarp for 1 week post‐fumigation. The field sites were subsequently prepared for planting by creating 15.25 cm high raised beds with 76.2 cm spacing between beds center‐to‐center. After installing drip irrigation, beds were covered with black plastic mulch. Sub‐surface drip irrigation was applied to maintain adequate soil moisture throughout the growing season. Fertilization was done via injection through the drip system with approximately 198 kg ha^‐1^ of nitrogen applied over the growing season.

The roots of each clone (bare‐root plant) were submerged in the *V. dahliae* Vd9602 inoculum suspension (1 × 10^6^ spores mL^‐1^) for 7–8 min before transplanting into a single row in the middle of planting beds. The spacing was 30.5 cm between plants within rows. The planting dates were October 31, 2016 (Global population), October 23, 2017 (Global and CA populations), and November 14, 2018 (CA population). The details of the experimental design for the Global population are detailed in Pincot et al. (2020). In 2018, the experiments were located side‐by‐side in the same field. We arranged accessions in square lattice experiment designs for the CA population with four single‐plant replications per accession (Hinkelmann & Kempthorne, 2005). There were four complete blocks (replications) in both years, with incomplete blocks arranged in randomized complete blocks (RCBs). We used 24 × 24 square lattices, with 24 accessions/incomplete block × 24 incomplete blocks/complete block × 4 complete blocks (2304 plants total). The R package *agricolae* (De Mendiburu & Simon, 2015) was used to assign accessions to incomplete blocks and randomize accessions within incomplete blocks.

### 4.4 SNP marker genotyping

DNA was isolated from newly emerged leaves harvested from field‐grown plants. Leaf samples were collected into 1.1 mL tubes or coin envelopes and freeze‐dried in a Benchtop Pro (VirTis SP Scientific, Stone Bridge, NY). We placed 0.2 g of dried leaf tissue/sample into wells of 2.0 mL 96‐well deep‐well plates. These tissue samples were ground using stainless steel beads in a Mini 1600 (SPEX Sample Prep, Metuchen, NJ). According to the manufacturer's instructions, genomic DNA (gDNA) was extracted from powdered leaf samples using the E‐Z 96®Plant DNA Kit (Omega Bio‐Tek, Norcross, GA). To enhance the quality and yield of the DNA and reduce polysaccharide carry‐through, the protocol was modified by adding Proteinase K to the lysis buffer to a final concentration of 0.2 mg mL^‐1^ and extending lysis incubation for 45 min. at 65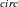 C. Once the lysate separated from the cellular debris, RNA was removed by adding RNase A and incubating the mixture at room temperature for 5 min before a final spin down. To ensure high DNA yields, the sample was incubated at 65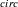 C for 5 min after adding elution buffer. DNA quantification was performed using Quantiflor dye (Promega, Madison, WI) on a Synergy HTX (Biotek, Winooski, VT). Training population accessions were genotyped with the Affymetrix®50K SNP Axiom®Array (Hardigan et al., 2020) on a GeneTitan™HT Microarray System at Affymetrix (Santa Clara, CA; https://www.thermofisher.com/order/CAtalog/product/551042?SID=srch‐srp‐551042) using gDNA samples that passed quality and quantity control standards. SNP genotypes were automatically called with the Affymetrix®Axiom®Analysis Suite software (v4.0.3.3, Affymetrix, Santa Clara, CA), followed by manual curation. All samples had a call rate greater than 89% and were retained for further analysis. The Affymetrix®Axiom®Analysis Suite and custom R scripts were used to identify and select SNP markers with well‐separated codominant genotypic clusters. The filtering and selection process yielded 25,865 high‐quality biallelic SNP markers for subsequent statistical analyses.

### 4.5 Disease resistance phenotyping

Each plant was phenotyped for resistance to VW by visually scoring the severity of symptoms on a 1‐to‐5 scale, where 1 = healthy and 5 = dead (Figure 1). Symptom severity was estimated using a combination of stunting, wilting, chlorosis, and dieback, as described by Gordon et al. (2006). The scores of the Global population, originally reported on a 1‐to‐9 scale (1 = healthy, 9 = dead) in Pincot et al. (2020), were linearly converted to 1 to 5. Phenotyping was initiated as soon as symptoms appeared and continued on a biweekly basis from May 30 to July 26 in 2016 (Global population), May 7 to June 29 in 2018 (Global and CA populations), and May 14 to July 27 in 2019 (CA population). This corresponded to 30 to 38 weeks post‐inoculation in 2017, 28 to 36 weeks post‐inoculation in 2018, and 26 to 36 weeks post‐inoculation in 2019. We weighted the disease AUDPS by the number of days of evaluation each year so that the minimum value is “1” and the maximum value is “5;” the same scale as for the final time point score. We used the progression of disease symptoms to determine when to initiate and terminate phenotyping. Symptoms had fully progressed and were most extreme among resistant and susceptible checks in the final time point (Score) in each year. We used the package *agricolae::audps()* to calculate the AUDPS for each replicate in each year (de Mendiburu, 2021; Simko & Piepho, 2012). Statistical analyses were applied to both the Score and AUDPS traits.

### 4.6 Statistical analyses

We used the R package *lme4::lmer()* for linear mixed model (LMM) analyses (Bates et al., 2015). As we did not observe an increase in efficiency in the lattice design relative to the RCB designs in either year, the data were analyzed using LMMs for RCB experiment designs with year, genotype, genotype × year, and residual effects (incomplete blocks were ignored) (Hinkelmann & Kempthorne, 2005). For within‐year analyses, the LMM is:

(1) $$y_{rgi}=Rep_{r}+Geno_{g}+\varepsilon_{rgi}$$

For between‐year analyses, the LMM is:

(2) $$\begin{array}{ccc} y_{regi} & = & {Year}_{e}+Year\;\times \;{Rep}_{er}+{Geno}_{g}\\ & & +\;Geno\;\times \;{Year}_{ge}+\varepsilon_{regi} \end{array}$$

where $y_{rgi}$ and $y_{regi}$ are the ith phenotypic observations of the gth genotype in the rth replicate in the eth year, $Rep_{r}$ is fixed effect of the rth replicate, $Year_{e}$ is the fixed effect of the eth year, $Geno_{g}$ is the random effect of the gth genotype, $Geno\times Year_{ge}$ is the random effect of the geth genotype by year interaction, and $\varepsilon_{rgi}$ and $\varepsilon_{regi}$ are the ith residual of the gth genotype in the rth replicate in the eth year, $Rep_{r}$ is fixed effect of the rth replicate.

Variance components for these effects were estimated using restricted maximum likelihood (REML). Heritability on a clone‐mean basis $H^{2}=V_{G}/V_{P}$ was estimated from REML estimates of the genotype ($V_{G}$), genotype × year ($V_{G\times T}$), and residual ($V_{\varepsilon }$) variance components, where $V_{P}=V_{G}+V_{G\times T}/t+V_{\varepsilon }/rt$, t is the number of years, and r is the harmonic mean of the number of replications per genotype. The harmonic means for the Global panel were r=3.75 in 2017, 3.77 in 2018, and 7.65 between years. The CA population harmonic means were r=3.77 in 2018, 4.01 in 2019, and 7.58 between years. The harmonic means include replicated checks. The EMMs for accessions were estimated for both Score and AUDPS using the R package *emmeans::emmeans()* (Lenth, 2021). EMMs were calculated within years (single‐year EMMs) or across years (cross‐year EMMs); for both single‐ and cross‐year EMMs, $Geno_{g}$ was treated as a fixed effect, and the year was included as a random effect for cross‐year calculations. These EMMs were used as dependent variables (phenotypic observations) for the genome‐wide association study (GWAS) and whole‐genome regression (WGR) models (Piepho et al., 2012). The phenotypic correlations between 2017 and 2018 (Global population) and 2018 and 2019 (CA population) EMMs for accessions were estimated using the R function *stats::cor.test()* (R Core Team, 2019).

### 4.7 Multivariate GWAS

The EMMs for individuals for each population were analyzed as separate dependant variables in a multivariate GWAS (MV‐GWAS) using GEMMA 0.98.1 (Zhou & Stephens, 2014). Some individuals were excluded due to missing genotypic or phenotypic records. The independent variables for these analyses were the genotypes for 27,111 Axiom^TM^ array SNP markers with alleles coded 0, 1, and 2 (Hardigan et al., 2020). The physical positions of these SNPs in the “UCD Royal Royce” reference genome are shown in File S3. The genomic relationship matrix (K) was estimated from the coded SNP marker genotypes as described by Pincot et al. (2020). The K matrix was used in MV‐GWAS analyses to correct for genetic relationships among individuals (Zhou & Stephens, 2014). We used a 5% false‐discovery rate (FDR)‐corrected significance threshold to test the null hypothesis of no SNP effect (Benjamini & Hochberg, 1995; Jiménez et al., 2022). Marker metadata (chromosome and position [bp]) are taken from the ‘UCD Royal Royce‘ FARR1 V1.0 reference genome (Hardigan et al., 2021), which is available on Phytozome (Goodstein et al., 2012) and the Genome Database for Rosaceae (GDR) (Jung et al., 2007).

### 4.8 Genomic prediction of breeding values

(GEBVs were estimated using the WGR methods of genomic best linear unbiased prediction (GBLUP), the Bayesian lasso (BL), and Bayes ridge regression (BRR). The input for these analyses was EMMs for accessions combined. Additionally, the two populations were combined; subsequently, the combined population was used to predict subsets of both the Global and CA populations. All analyses were performed using the R package *BGLR* with default parameter settings (Pérez & de Los Campos, 2014). Collectively, 14 studies were performed and yielded GEBV estimates for calculating genomic prediction accuracies for Score and AUDPS traits. To estimate the accuracy of genomic predictions with k=100 replications cross‐fold validation using seven different schemes. In scheme 1 (S1), 100 iterations of 80/20 CV using the combined population were analyzed. In scheme 2 (S2), 80% of the combined population was used to predict 20% of the population drawn exclusively from the elite panel for 100 iterations. In scheme 3 (S3), 80% of the combined population was used to predict 20% of the population drawn exclusively from the Global panel for 100 iterations. In scheme 4 (S4), 80% of the CA population was used to predict the remaining 20% of the CA population. In scheme 5 (S5), 80% of the CA population was used to predict the remaining 100% of the Global panel. In scheme 6 (S6), 80% of the Global panel was used to predict the remaining 100% of the elite panel. In scheme 7 (S7), 80% of the Global panel was used to predict the remaining 20% of the Global panel. Each scheme was replicated 100 times. A new training set is obtained to train the model in each realization. In schemes 4 and 6, the testing population does not change between iterations. The accuracy of genomic selection ($r_{\text{GS}}$) was estimated as the mean of correlations between observed phenotypes ($\bar{y}$) and GEBVs across the 100 replications, where the $\bar{y}$ are EMMs for accessions in the training population.

### 4.9 Genomic prediction of genetic variances and cross‐usefulness criteria

We used the approach described by Mohammadi et al. (2015) to estimate genomic‐estimated genetic variances for simulated segregating populations (200 individuals/population) arising from 423,660 crosses (a factorial design with reciprocals) among 437 individuals in the training population (prospective parents). The across‐year EMMs for disease severity score, Axiom^TM^ SNP array genotypes, and a reference genetic map developed for the cultivar “Camarosa” (Hardigan et al., 2020) were used as input in the R package *PopVar* to estimate population means (${\hat{\mu }}_{g}$) and genetic variances (${\hat{\sigma }}_{g}^{2}$) for each of the 423,660 simulated populations (Tiede & Neyhart, 2021). The cross‐usefulness criterion (U) was estimated by ${\hat{\mu }}_{g}-{\hat{\sigma }}_{g}$; smaller values are more desirable and correspond to a higher level of resistance.

## CONFLICT OF INTEREST STATEMENT

The authors declare that the research was conducted without any commercial or financial relationships that could be construed as a potential conflict of interest.

## AUTHOR CONTRIBUTIONS

**Mitchell J. Feldmann**: Conceptualization; data curation; formal analysis; investigation; methodology; project administration; resources; software; validation; visualization; writing original draft; writing review and editing. **Dominique D. A. Pincot**: Conceptualization; data curation; formal analysis; investigation; methodology; project administration; resources; software; validation; visualization; writing original draft; writing review and editing. **Mishi V. Vachev**: Data curation; formal analysis; investigation; methodology; resources; visualization; writing original draft; writing review and editing. **Randi A. Famula**: Data curation; methodology; project administration; resources; supervision; writing original draft; writing review and editing. **Glenn S. Cole**: Conceptualization; funding acquisition; investigation; project administration; resources; supervision; writing original draft; writing review and editing. **Steven J. Knapp**: Conceptualization; funding acquisition; investigation; methodology; project administration; resources; supervision; validation; visualization; writing original draft; writing review and editing.

## Supporting information


**Supplemental File 1** contains the estimated marginal means (EMMs) for the final time point (score) scores and the area under the disease progression stairs (AUDPS) for the accessions of the Global population in trial years 2017 and 2018 and the CA population in trial years 2018 and 2019.

**Supplemental File 2** contains the unfiltered genotypic data for m=25,865 SNPs from the FanaSNP Axiom Array (Hardigan et al., 2020) for all 921 entries evaluated in this study.

**Supplemental File 3** contains the results of multi‐trait genome‐wide association analyses for VW resistance (Score and AUDPS) in the diversity, elite, and combined populations.

## Data Availability

This study's phenotypic data, genotypic data, and GWAS summary statistics are available in the online version of this article.
